# Genetic Polymorphisms Contribute to the Individual Variations of Imatinib Mesylate Plasma Levels and Adverse Reactions in Chinese GIST Patients

**DOI:** 10.3390/ijms18030603

**Published:** 2017-03-13

**Authors:** Jing Liu, Zhiyu Chen, Hanmei Chen, Yingyong Hou, Weiqi Lu, Junyi He, Hanxing Tong, Yuhong Zhou, Weimin Cai

**Affiliations:** 1 Department of Clinical Pharmacy, School of Pharmacy, Fudan University, Shanghai 201203, China; nihaoliujing_001@126.com (J.L.); 14211030047@fudan.edu.cn (H.C.); 2 Department of Medical Oncology, Shanghai Cancer Center, Fudan University, Shanghai 200032, China; chanhj75@aliyun.com; 3 Department of Pathology, Zhongshan Hospital, Fudan University, Shanghai 200032, China; hou.yingyong@zs-hospital.sh.cn; 4 Department of General Surgery, Zhongshan Hospital, Fudan University, Shanghai 200032, China; lu.weiqi@zs-hospital.sh.cn (W.L.); doctorhjy@163.com (J.H.); thx121@163.com (H.T.); 5 Department of Medical Oncology, Zhongshan Hospital, Fudan University, Shanghai 200032, China

**Keywords:** imatinib mesylate, gastrointestinal stromal tumor, genetic polymorphisms, plasma levels, adverse reactions, Chinese

## Abstract

Imatinib mesylate (IM) has dramatically improved the outcomes of gastrointestinal stromal tumor (GIST) patients. However, the clinical responses of IM may considerably vary among single individuals. This study aimed to investigate the influences of genetic polymorphisms of drug-metabolizing enzyme (CYP3A4), transporters (ABCB1, ABCG2), and nuclear receptor (Pregnane X Receptor (PXR, encoded by *NR1I2*)) on IM plasma levels and related adverse reactions in Chinese GIST patients. A total of 68 Chinese GIST patients who have received IM 300–600 mg/day were genotyped for six single nucleotide polymorphisms (SNPs) (*CYP3A4 rs2242480*; *ABCB1 rs1045642*; *ABCG2 rs2231137*; *NRI12 rs3814055*, *rs6785049*, *rs2276706*), and the steady-state IM trough plasma concentrations were measured by a validated HPLC method. There were statistically significant variances in the steady-state IM trough plasma concentrations (from 272.22 to 4365.96 ng/mL). Subjects of *GG* in *rs2242480*, *T* allele carriers in *rs1045642* and *CC* in *rs3814055* had significantly higher steady-state IM dose-adjusted trough plasma concentrations. Subjects of *CC* in *rs3814055* had significantly higher incidence rate of edema. The genetic polymorphisms of *rs2242480*, *rs1045642*, *rs3814055* were significantly associated with IM plasma levels, and the genetic variations of *rs3814055* were significantly associated with the incidence rate of edema in Chinese GIST patients. The current results may serve as valuable fundamental knowledge for IM therapy in Chinese GIST patients.

## 1. Introduction

Gastrointestinal stromal tumors (GISTs) are epidemiologically analyzed to be the most common form of the mesenchymal tumor of the gastrointestinal tract, as the worldwide incidence and prevalence are estimated to be approximately 1 to 1.5 per 100,000 per year and 13 per 100,000, respectively [1]. At the present time, the combined application of surgery resection and molecular targeted drugs therapy is the most effective treatment for patients with the middle or high risk GISTs. Imatinib mesylate (IM) is a first-line targeted therapy for inoperable, metastatic, or recurrent *KIT*-positive GIST and for the adjuvant treatment of patients following resection of primary *KIT*-positive GIST [2]. After the clinical introduction of IM, the patient outcomes for GIST have been dramatically improved, with an impressive impact on both quality of life and long-term prognosis. However, there are large individual variations in clinical efficacy and adverse reactions of IM [3,4,5]. Ten to fifteen percent of patients who underwent IM treatment after 3–6 months progressed rapidly to widespread metastatic disease [6], about 30% of patients who developed serious adverse reactions had to stop taking medication [7]. Recently, the relationship between IM plasma concentrations and efficacy and toxicity have been described [8,9], minimal plasma concentration thresholds (1100 ng/mL) have been established, under which a substantial increase in treatment failure and drug resistance was observed [10]. Thus, inter-individual variations in IM pharmacokinetics may, therefore, have important clinical consequences. This implicates that timely monitoring of IM plasma concentration is warranted in GIST patients.

It has been found that genetic polymorphisms of main drug-metabolizing enzymes and transporters may significantly influence the inter-individual variations in drug reaction and disposition [11]. IM is orally administrated and was mainly metabolized in the liver by cytochrome P450 3A4 (CYP3A4), and effluxed by ATP Binding Cassette Subfamily B Member 1(ABCB1, P-glycoprotein), ATP Binding Cassette Subfamily G Member 2 (ABCG2, BCRP), etc. The current studies of individualized therapy for IM mainly focused on the genetic polymorphisms of main drug-metabolizing enzymes (CYP3A4) [12] and transporters (ABCB1, ABCG2) [13,14]. However, the variant differences in IM clinical effects and adverse reactions remains controversial, suggesting that there are some other genetic factors neglected. In addition, *CYP3A4 rs2242480*, the highest frequency single nucleotide polymorphisms (SNPs) in the Chinese population [15] was found to be correlated with the increased activity of CYP3A4, indicating that it is likely to be associated with IM pharmacokinetics.

Recently, nuclear receptors, as domain transcriptional regulators, have been found to play a crucial role in regulating the expression of relevant drug metabolic enzymes and transporters. Pregnane X Receptor (PXR, encoded by *NR1I2*), a member of the nuclear receptor superfamily, was increasingly found in simultaneously inducing the expression of CYP3A4 [16,17,18,19] and ABCB1 [17,18,20], predicting that the genetic polymorphisms of *NRI12* may have an effect on IM pharmacokinetics.

Based on these observations, six SNPs (*CYP3A4 rs2242480*; *ABCB1 rs1045642*; *ABCG2 rs2231137*; *NRI12 rs3814055*, *rs6785049*, *rs2276706*), which have been associated with the expression and/or function of the above drug-metabolizing enzyme and transporters genes and/or proteins in IM pharmacokinetics pathway, and with the minor allele frequency higher than 10% in Asian, were chosen as candidate SNPs, in order to investigate the influence of *CYP3A4*, *ABCB1*, *ABCG2* and *NR1I2* genetic polymorphisms on the steady-state IM dose-adjusted trough plasma concentrations and related adverse reactions in Chinese GIST patients. The results can be served as valuable fundamental knowledge for IM therapy, in order to make the antitumor treatment more successfully, and increase the safety and long-term tolerability of IM in Chinese GIST patients.

## 2. Results

### 2.1. Patients Characteristics and IM trough Plasma Concentrations

Sixty-eight Chinese GIST patients were enrolled, including 39 males and 29 females, whose characteristics and biological values are shown in Table 1. There are significant differences in IM trough plasma concentrations, and the mean IM trough plasma concentration of the study population is 1134.30 ng/mL, ranging from 272.22 to 4365.96 ng/mL (Table 2). About 47.06% of patients, whose IM trough plasma concentrations are under the minimal plasma concentration thresholds (1100 ng/mL), this may indicate a high risk of disease progression and treatment failure.

### 2.2. Genotype Frequencies

Frequencies of the six SNPs’ genotypes in the study population are shown in Table 3. The frequency expected for each genotype was evaluated on the basis of Hardy–Weinberg equilibrium. None of the observed SNPs’ frequencies was significantly different from the expected frequencies (*p* > 0.05), illustrating that all of the six SNPs’ frequencies in the study are in accordance with Hardy–Weinberg equilibrium. There is almost no linkage disequilibrium among all the six SNPs, and six SNPs in the study were in genetic equilibrium.

Comparisons of mutation frequencies for the six SNPs above, among different ethnic groups, are shown in Table 4. None of the observed SNPs’ frequencies in the study population were significantly different from the Healthy Han Chinese and Asian, but all six SNPs’ mutation frequencies were significantly different from Caucasian and African. These SNPs may contribute to inter-individual variations of IM reaction and disposition among different ethnic groups.

### 2.3. Effect of Genetic Polymorphisms of Drug-Metabolizing Enzyme CYP3A4 on IM Plasma Levels

**Figure 1:**
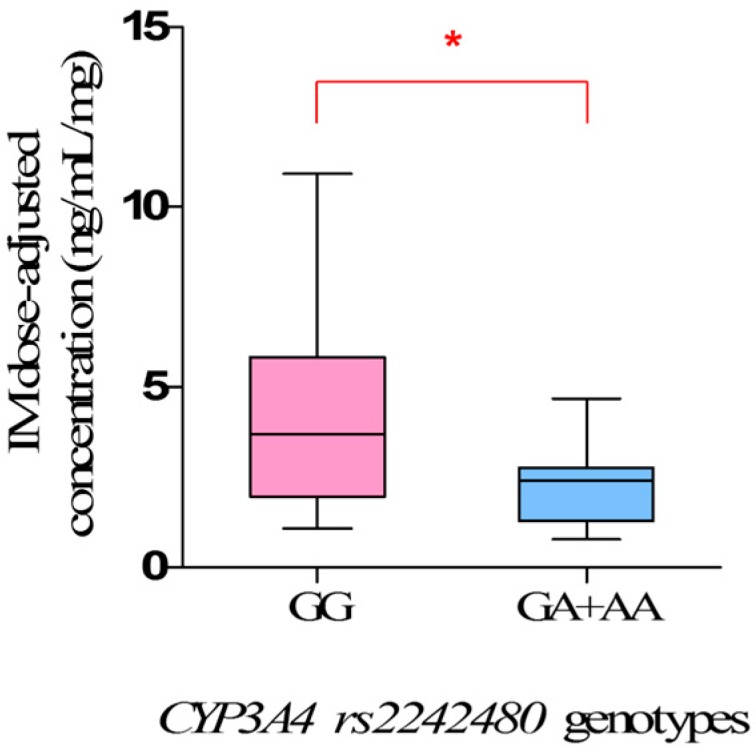
The steady-state Imatinib mesylate (IM) dose-adjusted trough plasma concentrations related to *CYP3A4 rs2242480* genotypes in 68 Chinese gastrointestinal stromal tumor (GIST) patients. (* *p* < 0.05).

For *CYP3A4 rs2242480*, the steady-state IM dose-adjusted trough plasma concentrations in mutant allele *A* carriers (*GA* + *AA*) (2.27 ± 0.32 ng/mL/mg) were significantly lower than that in wild-types (*GG*) (4.12 ± 0.40 mg/mL/mg) (*p* = 0.0171) (Figure 1, Table 5).

### 2.4. Effect of Genetic Polymorphisms of Transporters ABCB1/ABCG2 on IM Plasma Levels

**Figure 2:**
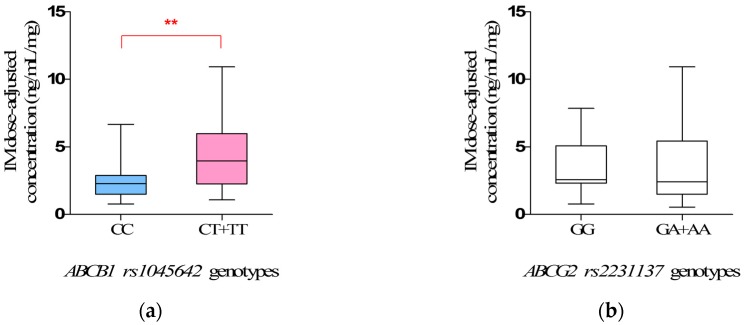
The steady-state Imatinib mesylate (IM) dose-adjusted trough plasma concentrations related to *ABCB1 rs1045642* (**a**) and *ABCG2 rs2231137* (**b**) genotypes in 68 Chinese gastrointestinal stromal tumor (GIST) patients (** *p* < 0.01).

For *ABCB1 rs1045642*, the steady-state IM dose-adjusted trough plasma concentrations in mutant allele *T* carriers (*CT + TT*) (4.36 ± 0.45 ng/mL/mg) were significantly higher than that in wild-types (*CC*) (2.56 ± 0.33 mg/mL/mg) (*p* = 0.0055) (Figure 2a, Table 6).

For *ABCG2 rs2231137*, there is no significant difference in the mean steady-state IM dose-adjusted trough plasma concentration in observed genotypes (*p* > 0.05) (Figure 2b, Table 6).

### 2.5. Effect of Genetic Polymorphisms of Nuclear Receptor PXR on IM Plasma Levels

**Figure 3:**
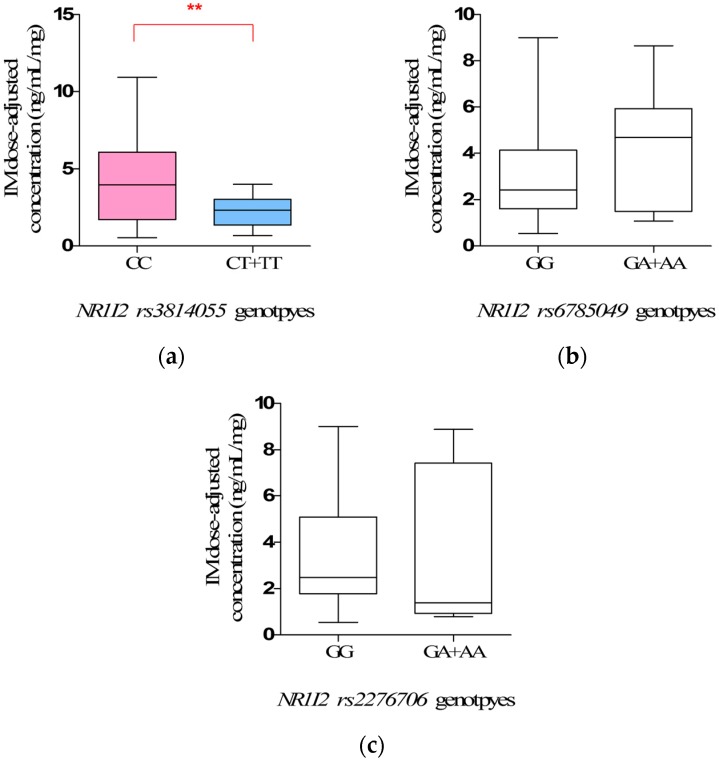
The steady-state Imatinib mesylate (IM) dose-adjusted trough plasma concentrations related to *NR1I2 rs3814055* (**a**); *rs6785049* (**b**); and *rs2276706* (**c**) genotypes in 68 Chinese gastrointestinal stromal tumor (GIST) patients (** *p* < 0.01).

For *NR1I2 rs3814055*, the steady-state IM dose-adjusted trough plasma concentrations in mutate allele *T* carriers (*CT + TT*) (2.34 ± 0.25 ng/mL/mg) were significantly lower than that in wild-types (*CC*) (4.26 ± 0.43 mg/mL/mg) (*p* = 0.0066) (Figure 3a, Table 7).

For *NR1I2 rs6785049* and *rs2276706*, there is no significant difference in the mean steady-state IM dose-adjusted trough plasma concentrations in observed genotypes (*p* > 0.05). (Figure 3b,c and Table 7).

### 2.6. Effect of Genetic Polymorphisms on IM Adverse Reactions

Among the 68 Chinese GIST patients, the most frequently observed adverse reactions related to IM include continuous edema, diarrhea, rash and myelosuppression.

**Figure 4:**
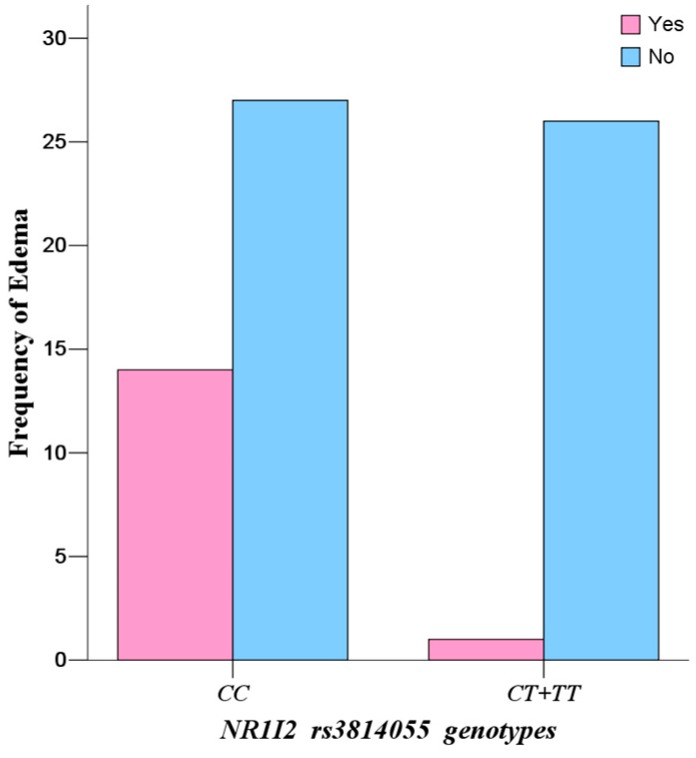
Comparison of the incidences of edema in 68 Chinese gastrointestinal stromal tumor (GIST) patients based on *NR1I2 rs3814055* polymorphisms.

For all six of the SNPs, only the mutation of *NR1I2 rs3814055* was significantly associated with the incidence rate of continuous edema in the study population. The incidence rate of continuous edema in wild-types (*CC*) (34.15%) in *NR1I2 rs3814055* was significantly higher than that in mutate allele *T* carriers (*CT + TT*) (3.70%) (*p* = 0.0030, Odds ratio (OR) = 13.48, 95% Confidence interval (CI): 1.65–109.98) (Figure 4, Table 8).

For the incidence rates of diarrhea, rash and myelosuppression, there are no significant differences in observed genotypes of all six SNPs (*p* > 0.05).

## 3. Discussion

In the past decade, imatinib mesylate, the first molecular-targeted drug with a known mechanism of efficacy, has radically changed the life expectancy of patients with GISTs. However, a large number of researchers have focused on the peculiar proto-oncogene of GISTs, and it can only partially explain the inter-individual variances of clinical response rate. While the germline DNA of patients remains important, as it dictates drug pharmacokinetics that may indirectly determine efficacy and toxicity. Based on these previous observations, we have involved the most relevant pharmacogenetic parameters, such as genetic polymorphisms of drug metabolizing enzymes, transporters and nuclear receptors, in order to investigate their influences on the steady-state IM dose-adjusted trough plasma concentrations and adverse reactions, for further explanation of the inter-individual variances on IM pharmacokinetics.

In our study, there was nearly a 16-fold variance in the steady-state IM trough plasma concentrations (from 272.22 to 4365.96 ng/mL) with IM doses ranging from 300 to 600 mg daily, which is much larger than the previous reports [10,23]. In addition, 47.06% of patients in the study, whose IM trough plasma concentrations had not reached the predefined minimal plasma concentration thresholds (1100 ng/mL), may be at the higher risk of disease progression and treatment failure.

Our study clearly showed the clinical impact of *CYP3A4 rs2242480* polymorphisms on IM plasma levels, and the steady-state IM dose-adjusted trough plasma concentrations in mutant allele *A* carriers (*GA* + *AA*) were significantly lower than that in wild-types (*GG*) (*p* = 0.0171). However, the influence of *rs2242480* polymorphisms on IM plasma levels in Chinese GIST patients has not been investigated previously. *rs2242480*, characterized by a G to A substitution in intron 10 of *CYP3A4*, is the most frequent SNP of *CYP3A4* in the Chinese population. The frequency of mutant allele *A* in the study was 17.58%, similar to Healthy Han Chinese and Asian [15,24], but significantly different from Caucasian (7.34%) and African (85.71%). The mutant allele *A* was reported to be associated with a higher CYP3A4 metabolic activity [25,26,27], thus increasing the clearance of IM, which was mainly metabolized by CYP3A4, leading the lower IM plasma levels. Therefore, the mutant allele *A* of *rs2242480* is a meaningful risk factor for predicting inadequate clinical efficacy of IM, and patients who carry mutant allele *A* of *rs2242480* may be suggested to have a higher dose therapy.

In addition, our study obviously showed the clinical impact of *ABCB1 rs1045642* polymorphisms on IM plasma levels, and the steady-state IM dose-adjusted trough plasma concentrations in mutate allele *T* carriers (*CT* + *TT*) were significantly higher than that in wild-types (*CC*) (*p* = 0.0066). Interestingly, the same association has been reported in Chronic Myelogenous Leukemia (CML) [28] patients under IM treatment, corroborating the importance of IM pharmacokinetics. The frequency of mutant allele *T* in the study was 36.76%, similar to Healthy Han Chinese and Asian [21], but significantly different from Caucasian (51.79%) and African (11.06%). The *ABCB1 rs1045642* mutant allele *T* was reported to reduce the transcript levels of *ABCB1* mRNA in vivo by as much as two- to four-fold when compared with allele *C* [29]. Thus, the mutant allele *T* was associated with a lower production of P-glycoprotein, leading to a lower drug clearance capability [30,31]. This might be responsible for the observation in the study that the mutant allele *T* carriers (*CT* + *TT*) have the higher IM plasma levels. We suggest that the mutant allele *T* of *rs1045642* is a significant risk factor for predicting excessive treatment of IM, and patients who carry the mutant allele *T* of *rs1045642* may be suggested to have a lower dose therapy.

However, the most interesting finding is the influence of *NR1I2 rs3814055* polymorphisms on IM plasma levels and adverse reaction. The steady-state IM dose-adjusted trough plasma concentrations in mutate allele *T* carriers (*CT* + *TT*) (2.34 ± 0.25 ng/mL/mg) were significantly lower than that in wild-types (*CC*), and the incidence rate of edema in mutate allele *T* carriers (*CT* + *TT*) was significantly lower than that in wild-types (*CC*) (*p* = 0.0030, OR = 13.48, 95% CI: 1.65–109.98). However, the influence of *rs3814055* polymorphisms on IM plasma levels and adverse reactions in Chinese GIST patients has not been reported previously, although a similar association has been reported in the studies on the pharmacokinetics of Tacrolimus in healthy subjects [32]. It is known that PXR is a crucial nuclear receptor to simultaneously regulate the expression of CYP3A4 [16,17,18,19] and ABCB1 [17,18,20]. *rs3814055*, located in 5′ Untranslated Regions of *NRI12*, is the highest frequency SNP of *NR1I2* in the Chinese population. The frequency of mutant allele *T* in the study was 23.53%, similar to Healthy Han Chinese and Asian [22], but significantly different from Caucasian (36.58%) and African (30.64%). It is reported that the erythromycin breath test in mutant allele *T* carriers (*CT* + *TT*) was two times stronger than wild-types (*CC*) by the inducing of rifampicin, indicated that mutant allele *T* was associated with a higher transcript levels of CYP3A4 mRNA and metabolic activity [33,34], thus increasing the clearance of IM, which was mainly metabolized by CYP3A4, leading to the lower IM plasma levels.

Continuous edema is the most common side effect of IM treatment, with incidence of 37.85% in the study population. Because severe and continuous edema (fluid retention) may result in interruption of IM, careful monitoring of severe edema is especially important in elderly patients (65 and older), patients with preexisting coronary artery disease or renal impairment and patients on higher doses of IM [35,36,37]. However, it is considered to be a concentration-dependent and dose-limiting adverse reaction of IM [38,39,40]—the higher the IM plasma concentrations, the higher the risk of incidence of the adverse reaction. This might be responsible for the observation in this study that the wild-types (*CC*) in *NR1I2 rs3814055* have higher IM plasma levels, leading to a high incidence rate of continuous edema. Therefore, allele *C* of *rs3814055* is a productive risk factor for predicting excessive treatment and high risk of adverse reaction of IM, and patients who carry the allele *C* of *rs3814055* may be suggested to have a lower dose or adjuvant diuretic therapy.

## 4. Materials and Methods

### 4.1. Subjects and Study Design

The study was performed according to the declaration of Helsinki and the International Conference on Harmonization-Good Clinical Practice standards. The study protocol was approved by the Ethics Committees of Zhongshan Hospital (ethical approval code: B2015-140R, 20151202) and the Shanghai Cancer Center (ethical approval code: 1604159-6, 20160425), affiliated with Fudan University. Details of the study were explained to all patients and informed consent was obtained. A total of 68 patients (39 males and 29 females) with newly diagnosed GIST were enrolled. All patients were orally administered 300–600 mg/day Imatinib Mesylate (Glivec, Novartis Pharma Stein AG, Basel, Switzerland) for at least one month. All of them were below 80 years of age, and had adequate hepatic and renal functions (Aspartate aminotransferase (AST)/Alanine aminotransferase (ALT) < 2× upper limit of normal (ULN), Total bilirubin in serum (TBIL) < 1.25 × ULN, Serum creatinine (Scr) < 1.5 × ULN). Patients who received medication known to affect IM plasma levels, such as verapamil, ketoconazole, and itraconazole, were excluded.

When IM plasma concentration reached a steady state (IM was regularly taken for 1 month at least), peripheral blood samples (0.5 h before IM dosing) of each GIST patient were drawn for IM trough plasma concentration determination and genotyping analysis, and the characteristics and biological values of the study population were recorded and summarized on the same day.

### 4.2. IM Trough Plasma Concentration Determination

Within 1 h of peripheral blood sample collection, plasma samples were prepared by centrifuging at 4000× *g* for 10 min at 4 °C, and a 100 µL aliquot of plasma was prepared by liquid–liquid extraction processing for IM trough plasma concentration determination, following a modified and validated HPLC method as reported earlier [41]. The HPLC system equipped with a Waters 1525 HPLC pump (Waters Corporation, Milford, MA, USA), a 2707 auto-sampler and a 2489 UV-detector linked to the Breeze™ Chromatography Data processing workstation (Waters Corporation, Milford, MA, USA) for recording and storing throughout analysis. Reversed phase HPLC analysis was carried out using a Symmetry C18 column (2.1 mm × 150 mm, 3.5 μm) (Waters Corporation, Milford, MA, USA) maintained at ambient temperature, with a mobile phase of Acetonitrile-20 mM ammonium acetate buffer (pH 6.8) (30:70, *v*/*v*), pumped at a flow rate of 0.2 mL/min and UV detection at a wavelength of 265 nm.

### 4.3. DNA Extraction and Genotyping

Total genomic DNA extraction was carried out using the method described previously [42]. The concentration and purity of extracted total genomic DNA samples were determined by a NANODROP LITE Spectrophotometer (Thermo Fisher Scientific Inc., Waltham, MA, USA). The genetic polymorphisms of *CYP3A4 rs2242480*, *ABCB1 rs1045642*, *ABCG2 rs2231137* were determined by using polymerase chain reaction (PCR) restriction fragment length polymorphism (RFLP) method, while the genetic polymorphisms of *NR1I2 rs3814055*, *rs6785049*, *rs2276706* were determined by using PCR and then sequencing. Analyses were performed using an Applied Biosysterms 2707 Thermal Cycler (Applied Biosystems, Foster, CA, USA). Sequencings were carried out on Thermo Fisher Scientific Inc. USA. Genotyping accuracy was confirmed by sequencing for two cases of each genotype. The PCR primers and reaction conditions were listed in the Supplementary Materials section (Tables S1–S3).

### 4.4. Adverse Reactions Monitoring

All patients were followed up in special clinics for GISTs to monitor IM-induced adverse reactions, which were evaluated according to the National Cancer Institute Common Terminology Criteria for Adverse Events version 4.0 (NCI CTCAE v4.0).

### 4.5. Statistical Analysis

All statistical analyses were performed with SPSS^®^ software, version 21.0 (IBM, Chicago, IL, USA). The results are expressed as mean ± SD, all of the tests were two-sided, and two-sided *p* < 0.05 was considered as statistically significant.

The Hardy–Weinberg equilibrium test was performed using an appropriate χ^2^-test. Linkage disequilibrium (LD) based association analysis was measured using the online software SHEsis (Bio-X Life Science Research Center, Shanghai, China) [43]. The statistical differences of IM dose-adjusted trough plasma concentration between genotypes of each SNP were analyzed by Mann-Whitney or Kruskal-Wallis tests. The statistical differences of IM adverse reactions between genotypes of each SNP were analyzed by chi-square or Fisher’s exact tests.

## 5. Conclusions

In summary, our findings indicate that the genetic polymorphisms of *CYP3A4*, *ABCB1* and *NR1I2* may make an important contribution to IM plasma levels and related adverse reactions. Because of the limited sample size of the study, further research should be carried out to verify the associated genetic polymorphisms on IM plasma levels and adverse reactions. The current results may serve as valuable fundamental knowledge for IM therapy in Chinese GIST patients.

## Figures and Tables

**Table 1:** Characteristics and biological values of 68 Chinese GIST patients on the IM plasma concentration determination day.

Characters (Unit)	Mean ± SD (Range)
**Patient characteristics**	
Number of recipients	68
Age (years)	56.11 ± 0.50 (29–78)
Gender (male/female)	39/29
Height (cm)	166.00 ± 7.04 (150.00–182.00)
Weight (kg)	61.51 ± 9.47 (45.00–85.00)
BMI	22.18 ± 3.25 (15.09–28.65)
**Biological values**	
White blood cell count (×10^9^/L)	5.37 ± 1.42 (2.01–12.40)
Red blood cell count (×10^12^/L)	3.70 ± 0.51 (2.42–5.10)
Neutrophil count (×10^9^/L)	3.19 ± 1.30 (0.50–11.50)
Hemoglobin (g/L)	117.83 ± 12.00 (46.00–152.00)
Platelet (×10^9^/L)	187.00 ± 66.57 (29.00–332.00)
Alanine aminotransferase (U/L)	30.17 ± 11.28 (5.00–73.00)
Aspartate aminotransferase (U/L)	31.67 ± 4.75 (12.80–77.00)
Albumin (g/L)	40.60 ± 4.68 (24.00–51.90)
Serum creatinine (μmol/L)	77.00 ± 14.13 (37.00–133.00)
Uric acid (μmol/L)	227.80 ± 71.55 (96.00–512.00)
Blood urea nitrogen (mmol/L)	5.65 ± 1.50 (1.70–202.00)

Abbreviations: GIST, Gastrointestinal stromal tumor; IM, Imatinib mesylate; BMI: Body Mass Index = Weight/(Height/100)^2^; SD, Standard deviation.

**Table 2:** The steady-state IM trough plasma concentrations in 68 Chinese GIST patients.

Characters (Unit)	Mean ± SD (Range)
Number of total patients	68
IM trough plasma concentrations (ng/mL)	1134.30 ± 1141.69 (272.22–4365.96)
IM dose-adjusted trough plasma concentrations (ng/mL/mg)	3.71 ± 2.53 (0.68–10.91)
Number of patients (<1100 ng/mL)	32 (47.06%)
IM trough plasma concentrations (ng/mL)	713.86 ± 239.76 (272.22–1095.20)
IM dose-adjusted trough plasma concentrations (ng/mL/mg)	1.80 ± 0.61 (0.68–2.74)
Number of patients (>1100 ng/mL)	36 (52.94%)
IM trough plasma concentrations (ng/mL)	2339.81 ± 840.04 (1118.31–4365.96)
IM dose-adjusted trough plasma concentrations (ng/mL/mg)	6.03 ± 2.04 (2.80–10.91)

Abbreviations: IM, Imatinib mesylate; GIST, Gastrointestinal stromal tumor; SD, Standard deviation.

**Table 3:** Frequencies of six SNPs in 68 Chinese GIST patients.

SNP_ID	Gene	Genotype	*n*	Identified Frequency (%)	Allele	Allele Frequency (%)	HWE
							*p*-Value
*rs2242480*	*CYP3A4*	*GG*	48	70.59	*G*	82.35	0.12
		*GA*	16	23.53	*A*	17.65	
		*AA*	4	5.88			
*rs1045642*	*ABCB1*	*CC*	29	42.64	*C*	63.24	0.52
		*CT*	28	41.18	*T*	36.76	
		*TT*	11	16.18			
*rs2231137*	*ABCG2*	*GG*	28	41.18	*G*	63.97	0.99
		*GA*	31	45.59	*A*	36.03	
		*AA*	9	13.23			
*rs3814055*	*NR1I2*	*CC*	41	60.30	*C*	76.47	0.60
		*CT*	22	32.35	*T*	23.53	
		*TT*	5	7.35			
*rs6785049*	*NR1I2*	*GG*	21	30.88	*G*	55.88	0.99
		*GA*	34	50.00	*A*	44.12	
		*AA*	13	19.12			
*rs2276706*	*NR1I2*	*GG*	39	57.35	*G*	75.00	0.84
		*GA*	24	35.30	*A*	25.00	
		*AA*	5	7.35			

Abbreviations: SNPs, single nucleotide polymorphisms; GIST, Gastrointestinal stromal tumor; *n*, the numbers of patients; HWE, Hardy-Weinberg equilibrium.

**Table 4:** Comparisons of mutation frequencies for six SNPs among different ethnic groups.

SNP	Mutation Frequency (%)													
	Study Population		Healthy Han Chinese			Asian			Caucasian			African		
	*n*	Freq. ^a^	*n*	Freq. ^a^	Ref. ^b^	*n*	Freq. ^a^	Ref. ^b^	*n*	Freq. ^a^	Ref. ^b^	*n*	Freq. ^a^	Ref. ^b^
*rs2242480*	68	17.68	976	22.10	[15]	120	20.00	NCBI	218	7.34 **	NCBI	224	85.71 ^c,^**	NCBI
*rs1045642*	68	36.76	208	40.40	[21]	120	43.33	NCBI	226	51.79 **	NCBI	226	11.06 ^c,^**	NCBI
*rs2231137*	68	36.03	90	28.89	NCBI	120	29.55	NCBI	120	1.67 **	NCBI	120	5.00 ^e,^**	NCBI
*rs3814055*	68	23.53	286	21.80	[22]	120	22.50	NCBI	226	36.58 **	NCBI	226	30.64 ^d^	NCBI
*rs6785049*	68	44.12	300	37.00	[22]	172	51.67	NCBI	226	62.33 **	NCBI	226	3.56 **	NCBI
*rs2276706*	68	25.00	286	21.80	[22]	120	22.50	NCBI	120	40.00 **	NCBI	116	27.59 ^c^	NCBI

^a^ Frequency; ^b^ Reference; ^c^ Sub-Saharan African; ^d^ African-American; ^e^ Yoruba in Ibadan; ** *p* < 0.01. Abbreviations: SNPs, single nucleotide polymorphisms; *n*, the numbers of patients.

**Table 5:** Association of *CYP3A4 rs2242480* genotypes with the steady-state IM dose-adjusted concentrations in 68 Chinese GIST patients.

SNP_ID	Gene	Genotype	*n*	IM Dose-Adjusted Concentration (ng/mL/mg)	
				Mean ± SD	*p*
*rs2242480*	*CYP3A4*	*GG*	48	4.12 ± 0.40	0.0171 *
		*GA + AA*	20	2.27 ± 0.32	

Abbreviations: IM, Imatinib mesylate; GIST, Gastrointestinal stromal tumor; SNP, Single nucleotide polymorphism; *n*, the numbers of patients; SD, Standard deviation. * *p* < 0.05.

**Table 6:** Association of *ABCB1/ABCG2* polymorphisms with the steady-state IM dose-adjusted concentrations in 68 Chinese GIST patients.

SNP_ID	Gene	Genotype	*n*	IM Dose-Adjusted Concentration (ng/mL/mg)	
				Mean ± SD	*p*
*rs1045642*	*ABCB1*	*CC*	29	2.56 ± 0.33	0.0055 **
		*CT + TT*	39	4.36 ± 0.45	
*rs2231137*	*ABCG2*	*GG*	28	3.51 ± 0.40	0.7158
		*GA + AA*	40	3.76 ± 0.50	

Abbreviations: IM, Imatinib mesylate; GIST, Gastrointestinal stromal tumor; SNP, Single nucleotide polymorphism; *n*, the numbers of patients; SD, Standard deviation. ** *p* < 0.01.

**Table 7:** Association of *NR1I2* polymorphisms with the steady-state IM dose-adjusted concentrations in 68 Chinese GIST patients.

SNP_ID	Gene	Genotype	*n*	IM Dose-Adjusted Concentration (ng/mL/mg)	
				Mean ± SD	*p*
*rs3814055*	*NR1I2*	*CC*	41	4.26 ± 0.43	0.0066 **
		*CT + TT*	27	2.34 ± 0.25	
*rs6785049*	*NR1I2*	*GG*	21	3.17 ± 0.33	0.2010
		*GA + AA*	47	4.09 ± 0.73	
*rs2276706*	*NR1I2*	*GG*	39	3.55 ± 0.32	0.9556
		*GA + AA*	29	3.62 ± 1.62	

Abbreviations: IM, Imatinib mesylate; GIST, Gastrointestinal stromal tumor; SNP, Single nucleotide polymorphism; *n*, the numbers of patients; SD, Standard deviation. ** *p* < 0.01.

**Table 8:** Comparison of the incidences of edema related to *NR1I2 rs3814055* polymorphisms in 68 Chinese GIST patients.

SNP_ID	Gene	Genotype	*n*	Edema				
				Yes (%)	No (%)	*p*	OR	95% CI
*rs3814055*	*NR1I2*	*CC*	41	14 (34.15)	27 (65.85)	0.0030 **	13.48	1.65, 109.98
		*CT + TT*	27	1 (3.70)	26 (96.30)			

Abbreviations: GIST, Gastrointestinal stromal tumor; SNP, Single nucleotide polymorphism; *n*, the numbers of patients; OR, Odds ratio; CI, Confidence interval. ** *p* < 0.01.

## Supplementary Materials

Supplementary materials can be found at www.mdpi.com/1422-0067/18/3/603/s1.

## Abbreviations

ABCB1	ATP-binding cassette (ABC) transporters subfamily B member 1
ABCG2	ATP-binding cassette (ABC) transporters subfamily G member 2
ALT	Alanine aminotransferase
AST	Aspartate aminotransferase
CI	Confidence interval
CYP3A4	Cytochrome P450 3A4
GIST	Gastrointestinal stromal tumor
HPLC	High Performance Liquid Chromatography
HWE	Hardy–Weinberg equilibrium
IM	Imatinib mesylate
OR	Odds ratio
PCR	Polymerase chain reaction
PXR	Pregnane X Receptor
RFLP	Restriction fragment length polymorphism
Scr	Serum creatinine
SNP	Single nucleotide polymorphism
TBIL	Total bilirubin in serum
ULN	Upper limit of normal
