# Supplementary material for: Genetic Polymorphisms Contribute to the Individual Variations of Imatinib Mesylate Plasma Levels and Adverse Reactions in Chinese GIST Patients
Source: Int J Mol Sci. 2017 Mar 13;18(3):603. doi: 10.3390/ijms18030603 (PMC5372619; doi:10.3390/ijms18030603)
Supplement: Supplementary file 1 [file ijms-18-00603-s001.pdf]

Genetic Polymorphisms Contribute to the Individual Variations of Imatinib Mesylate Plasma Levels and Advers e Reactions in Chinese GIST Patients

Jing Liu , Zhiyu Chen, Hanmei Chen, Yingyong Hou, Weiqi Lu, Junyi He, Hanxing Tong, Yuhong Zhou and Weimin Cai

Table S1. Primers for polymerase chain reaction (PCR).

SNP_ID	Gene	Primers (5′-3′; F = forward, R = reverse)	AMP_LEN (bp)
rs2242480	*CYP3A4*	*F: CAC CCT GAT GTC CAG CAG AAA CT*<br>*R: AAT AGA AAG CAG ATG AAC CAG AGC C*	**287**
rs1045642	*ABCB1*	*F: TGC TGG TCC TGA AGT TGA TCT GTG AAC*<br>*R: ACA TTA GGC AGT GAC TCG ATG AAG GCA*	**248**
rs2231137	*ABCG2*	*F: CTC ATC CAT CCT TTT CCT GCT T*<br>*R: AGC CAC TCT TCA GTT TTA CTC G*	**483**
rs3814055	*NR1I2*	*F: CCC AGC AGT GAG CTG TGT AA*<br>*R: AGC TGA GGG CTC TTT CCT CT*	**645**
rs6785049	*NR1I2*	*F: GGT TGT GAG GGG AGA GAT GA*<br>*R: AGC CAC CTG TGG ATG GTA AC*	**526**
rs2276706	*NR1I2*	*F: CCC CTT TTC CTG TGT TTT TG*<br>*R: CAA CAT TAA GTG ATT GTT TTC ATG C*	**637**

Table S2. Primers for polymerase chain reaction (PCR).

**Gene**	*CYP3A4*		*ABCB1*		*ABCG2*		*NR1I2*			
**SNP**	***rs2242480***		***rs1045642***		***rs2231137***		***rs3814055***<br>***rs6785049***		***rs2276706***	
**Pre-denature**	94 ℃	5 min	94 ℃	5 min	95 ℃	3 min	94 ℃	5 min	94 ℃	5 min
**Denature**	94 ℃	30 s	94 ℃	30 s	95 ℃	30 s	94 ℃	30 s	94 ℃	30 s
**Annealing**	62 ℃	30 s	60 ℃	30 s	58 ℃	30 s	58 ℃	30 s	58 ℃	30 s
**Extension**	72 ℃	1 min	72 ℃	1 min	72 ℃	50 s	72 ℃	1 min	72 ℃	1 min
**Cycles**	30		30		35		30		35	
**Extension**	72 ℃	7 min	72 ℃	7 min	72 ℃	10 min	72 ℃	7 min	72 ℃	7 min
**Storage**	4 ℃	∞	4 ℃	∞	4 ℃	∞	4 ℃	∞	4 ℃	∞

Table S3. Restriction endonuclease digestion conditions.

**Gene**	***CYP3A4***	***ABCB1***	***ABCG2***
**SNP**	***rs2242480***	***rs1045642***	***rs2231137***
**Reaction Volume (μL)**	20	20	20
**Restriction Enzyme (μL)**	Rsa I, 1	Mbo I, 0.5	TspR I, 0.5
**Rraction Buffer (μL)**	10x Buffer Tango, 2<br>BSA, 2	10× Buffer R, 2	10x Buffer Tango, 2
**PCR product (μL)**	10	10	10
**double-distilled H2O (μL)**	5	7.5	7.5
**Incubation Temperature (℃)**	37	37	37
**Incubation Time (h)**	3	3	3
**Agarose Gel Electrophoresis (%)**	2	2	2
